# Evaluating Monthly Flow Prediction Based on SWAT and Support Vector Regression Coupled with Discrete Wavelet Transform

**DOI:** 10.3390/w14172649

**Published:** 2022-08-27

**Authors:** Lifeng Yuan, Kenneth J. Forshay

**Affiliations:** 1 U.S. Environmental Protection Agency, Center for Environmental Solutions and Emergency Response, Robert S. Kerr Environmental Research Center, Ada, OK 74820, USA; 2 U.S. Environmental Protection Agency, Center for Environmental Solutions and Emergency Response, Homeland Security Materials Management Division, Durham, NC 27711, USA

**Keywords:** SWAT, support vector regression, streamflow prediction, wavelet transform, Illinois River watershed

## Abstract

Reliable and accurate streamflow prediction plays a critical role in watershed water resources planning and management. We developed a new hybrid SWAT-WSVR model based on 12 hydrological sites in the Illinois River watershed (IRW), U.S., that integrated the Soil and Water Assessment Tool (SWAT) model with a Support Vector Regression (SVR) calibration method coupled with discrete wavelet transforms (DWT) to better support modeling watersheds with limited data availability. Wavelet components of the simulated streamflow from the SWAT-Calibration Uncertainty Procedure (SWAT-CUP) and precipitation time series were used as inputs to SVR to build a hybrid SWAT-WSVR. We examined the performance and potential of the SWAT-WSVR model and compared it with observations, SWAT-CUP, and SWAT-SVR using statistical metrics, Taylor diagrams, and hydrography. The results showed that the average of *RMSE*-observation’s standard deviation ratio (*RSR*), Nash–Sutcliffe efficiency (*NSE*), percent bias (*PBIAS*), and root mean square error (*RMSE*) from SWAT-WSVR is 0.02, 1.00, −0.15, and 0.27 m^3^ s^−1^ in calibration and 0.14, 0.98, −1.88, and 2.91 m^3^ s^−1^ in validation on 12 sites, respectively. Compared with the other two models, the proposed SWAT-WSVR model possessed lower discrepancy and higher accuracy. The rank of the overall performance of the three SWAT-based models during the whole study period was SWAT-WSVR > SWAT-SVR > SWAT-CUP. The developed SWAT-WSVR model supplies an additional calibration approach that can improve the accuracy of the SWAT streamflow simulation of watersheds with limited data.

## 1. Introduction

A precise and reliable monthly streamflow prediction model is helpful in the planning, management, development, and protection of water resources, such as future flood and drought forecasting, reservoirs, and/or agricultural water management [1–3]. However, many hydrological elements (e.g., precipitation, runoff, sediment, flood, and streamflow) have highly complex, nonlinear, non-stationary, and uncertainty features, which is a challenge for conventional hydrological methods when analyzing and predicting the complex patterns and inherent variabilities of rainfall–runoff relationships [4–8]. Hence, accurate rainfall–runoff prediction became a difficult task in stochastic hydrology; subsequently, new theories and methods, such as machine learning methods [9,10], have been introduced to improve rainfall–runoff forecasting.

Support vector machine (SVM) is a machine learning method, which can be considered a data-driven black-box model [11]. SVM focuses on determining a kernel function and searching for an optimum separating hyperplane based on the kernel function selected. This separating hyperplane determines an optimal parameter combination fitting of the observations. Meanwhile, the search process avoids overfitting and make SVM present better generalization characteristics [12]. Compared to an artificial neural network (ANN), SVM performs better [13] in some hydrological applications because it applies a structural risk minimization principle to obtain a global optimum solution rather than a solution based on the empirical risk minimization as applied in ANN [4,13–15]. SVM can solve the nonlinear problem in a low dimension input space by projecting to a higher dimension feature space where an original nonlinear problem is converted into a linear problem [16,17]. SVM has been widely applied in recent decades to hydrological prediction worldwide. For instance, Shabri and Suhartono (2012) [18] used the least-squares SVM (LSSVM) method to predict streamflow in Peninsular Malaysia to compare performance of different models’ including LSSVM, autoregressive integrated moving average (ARIMA), ANN, and regular SVM. Kalteh (2013) [4] compared the prediction accuracy of monthly flow discharge from an artificial neural network (ANN) method and the support vector regression (SVR) model coupled with a wavelet transform in two stations in northern Iran. Chiogna et al. (2018) [19] combined the Soil and Water Assessment Tool (SWAT) model and SVR method to predict hydropeaking for the Upper Adige River watershed in northeast Italy and applied a wavelet method to analyze the price of energy. However, these studies only used a few hydrological stations to test their methods and evaluate the model performance. Nourani et al. (2015) [20] proposed a two-stage SVM method with spatial statistics to simulate monthly river suspended sediment load for 15 sites within the Ajichay River in northwest Iran. Yuan and Forshay (2021) [21] developed a seasonal SWAT model coupled with SVR for 13 hydrological stations using a spatial calibration method to improve the accuracy of monthly streamflow prediction in the Illinois River watershed. From these efforts, it is clear that the SVM method has powerful predictive ability depending on calibration datasets and can accurately capture nonlinear relationships between the input and output variables. However, the SVM prediction method neglects the detailed characteristics and processes of a watershed system [22] and simplifies the complexity of the rainfall–runoff relationship [23].

Wavelet analysis is a mathematical function with an auto-adaptive time-frequency window (i.e., the width of time and frequency may change) and is suited to analyze and calculate stationary or non-stationary time series signals [4]. In the high-frequency period of signals, the size of a frequency window becomes larger while the size of a time window becomes smaller and otherwise occurs in the signals low-frequency period. Wavelet analysis has an excellent capability to reduce data noise, analyze variabilities, periodicities, and trends of hydrological time series and is suitable for handling the non-stationary flow signals [24,25]. To date, the wavelet analysis method has been widely applied in hydrological prediction. For example, Nourani et al. (2014) [7] reviewed applications of hybrid wavelet-Artificial Intelligence (AI) models in hydrology and presented remarkable progress when integrating wavelet analysis and AI models to improve the prediction accuracy of hydrologic models in the recent couple of decades. Noraini and Norhaiza (2017) [13] compared different wavelet denoising techniques and decomposition levels, and input streamflow time series after wavelet denoising into SVR based on a radial basis function (RBF) kernel to improve 1-month-ahead streamflow prediction in the Segamat River basin in southern Malaysia. Sun et al. (2019) [26] integrated multiple methods, such as the autoregressive model, autoregressive moving average (ARMA) model, ANN model, and linear regression (LR) model, with wavelet transform and compared their differences in performance while predicting daily streamflow in the Heihe River basin of northern China, where they found that the wavelet-based method can effectively improve streamflow simulations compared to other models. Nalley et al. (2020) [27] used several wavelet-transform-based methods to improve the performance of extending streamflow records for areas in Canada with limited data available. From the works mentioned above, it was inferred that wavelet transform typically worked as a data pre-processing tool before employing conventional hydrological analysis methods or data-driven models and could raise the accuracy of the flow prediction. The main concerns regarding wavelet applications in hydrological forecasting are the proper selection of the mother wavelet function and the determination of decomposition levels corresponding to the specific hydrological time series [7,28]. Integrating these machine learning methods with physically based hydrologic models could better describe streamflow by incorporating the constraints of the physical world that include advanced mathematical techniques to discover the complex hidden or obscured signals that are difficult or impossible to model in a physically based modeling system.

Many physically based hydrologic models have been developed and applied to predict streamflow [29]. Among these models, the Soil and Water Assessment Tool (SWAT) is a conceptual, physically based, watershed-scale hydrologic model that has been extensively applied worldwide [30]. SWAT takes a large number of physical processes of hydrology into account. Hence, it requires extensive data and parameter inputs. Often, data are unavailable in certain regions due to time or economic cost or limitations of measurement technologies, especially in some developing countries. Therefore, the unknown values of many parameters in SWAT can only be determined via the procedure of calibration [3]. Calibration is a time-consuming and complicated process since it involves parameterization, optimal algorithm determination, and extensive iterative computation to find optimal value ranges and parameter combinations [11,31,32].

Moreover, the issue of parameter non-uniqueness is that different parameter sets might produce very similar simulated signals with the observed flow time series, which makes effective calibration harder to achieve [31]. To obtain appropriate parameter combinations, it requires researchers to have a profound understanding of hydrological parameters and processes and familiarity of local hydrological conditions and physical features in a study area. To raise the accuracy of hydrological models prediction, especially for a region with limited data available, several efforts have evaluated the performance and potential of SWAT coupling with the SVR methods in streamflow prediction [11,15,33,34], yet few efforts [19,35] have attempted to couple a distributed physically based model and a machine learning method to improve the rainfall–runoff simulation. In some cases, we do not achieve a desirable result of flow prediction with acceptable accuracy, even after conducting comprehensive model calibration. Here, we attempt to improve the accuracy of a commonly used physically based model (SWAT) by integrating discrete wavelet transform functions and support vector machines in a system with complex non-linear rainfall–runoff relationships to support modeling efforts in watersheds with limited data.

The object of this study aims to show how the SVM method with wavelet transforms can be used to improve the monthly flow prediction of the calibrated SWAT hydrological model in the Illinois River watershed (IRW), U.S. This work studied and compared the performance of calibrated SWAT (SWAT-CUP), regular SWAT-SVR without applying wavelet transform, and SWAT-WSVR coupled with wavelet transform for monthly flow prediction for 12 hydrological stations in the IRW. This study is helpful to improve streamflow prediction at a month scale in some areas with sparse data.

## 2. Methodology

**Figure 1.**
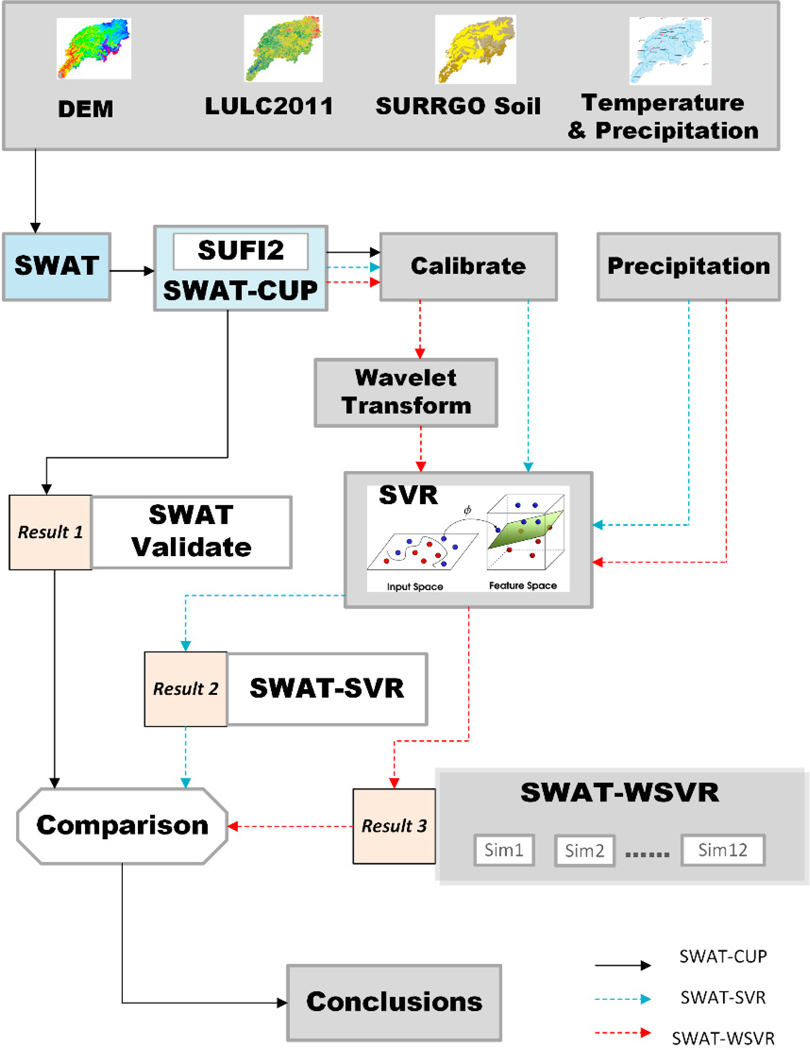
The developing flowchart of different models: SWAT-CUP, SWAT-SVR, and SWAT-WSVR.

To improve monthly flow prediction, we developed the hybrid model SWAT-WSVR based on SWAT and SVR with discrete wavelet transforms. First, we developed SWAT models for twelve sites after SWAT-CUP (SWAT Calibration and Uncertainty Program) calibration progresses. Then, the flow at month *t* simulated by SWAT-CUP calibration and corresponding precipitation (i.e., applying the Thiessen polygons divide method to allocate the NCDC meteorological stations to the USGS hydrological sites) of each site served as SVR input variables to predict flow on month *t*. It was a regular SWAT-SVR model. Next, the simulated monthly flow from SWAT-CUP was decomposed using the discrete wavelet transform (DWT) to obtain wavelet coefficients and approximation at different scale resolutions, which were served as inputs of SWAT-WSVR with precipitation data. The results from SWAT-CUP worked as a benchmark compared with the results from SWAT-SVR and SWAT-WSVR. To estimate the performance of different models, we used a metric such as the Nash–Sutcliffe efficiency (*NSE*), Percent Bias (*PBIAS*), Root Mean Square Error (*RMSE*), and *RMSE*-observations standard deviation ratio (*RSR*) to estimate the model results quantitatively. Finally, we combined calibrated (or training) and validated (or testing) time series data and re-estimated the entire model performance based on Pearson’s correlation coefficient (*r*), *RMSE*, and normalized standard deviation (*NSD*) and plotted the Taylor diagram and hydrography to compare their performance difference graphically. Figure 1 showed the construction flowchart of three hydrology models.

### 2.1. Watershed Description and Data Source

**Figure 2.**
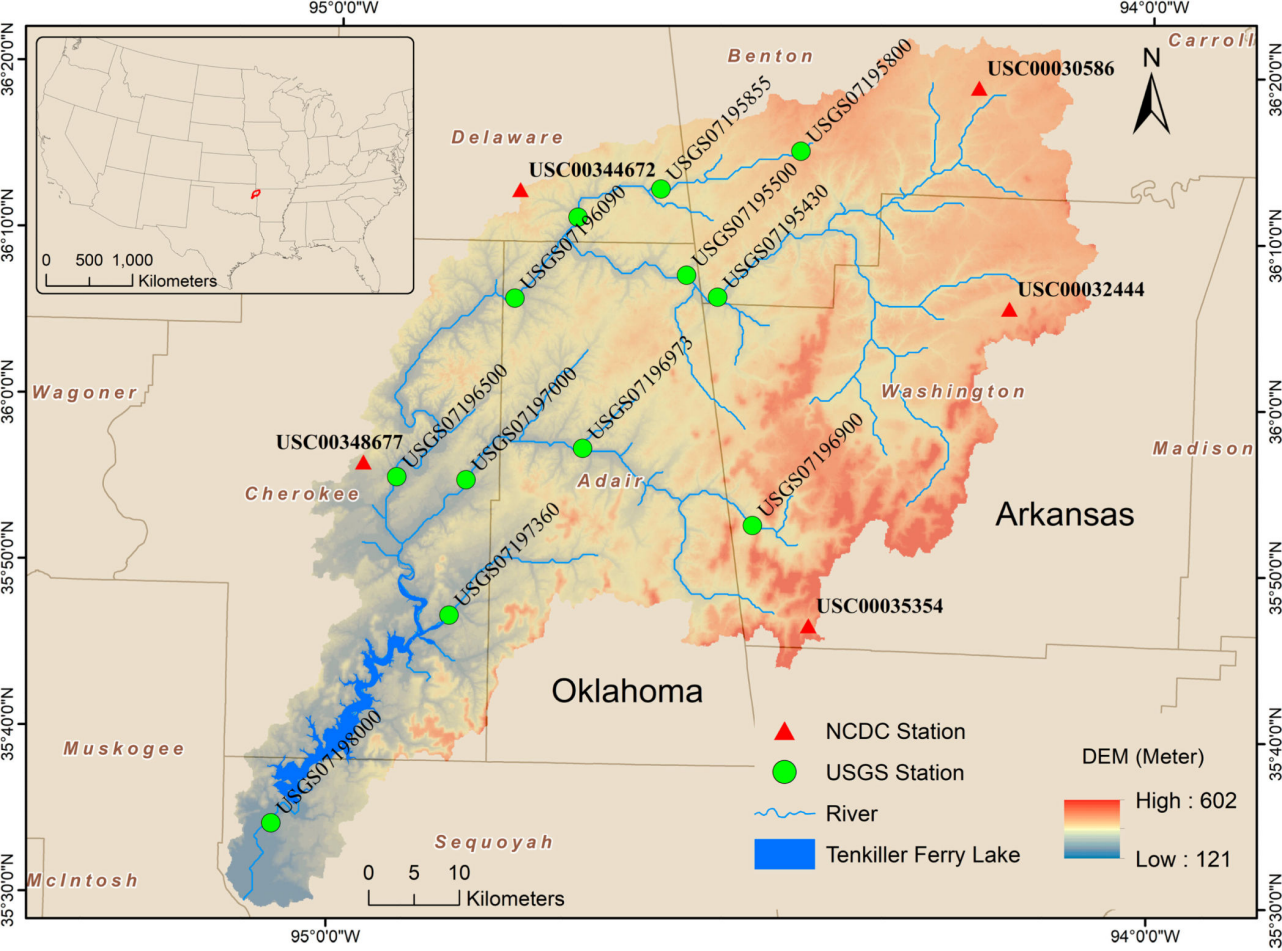
The geographic distribution of NCDC meteorological stations, USGS hydrological stations, lakes, and rivers in the IRW.

The IRW (35°31^’^–36°9^’^ N, 94°12^’^–95°2^’^ W) covers about a 4200 km^2^ drainage area and crosses Arkansas and Oklahoma, U.S. The average basin slope is about 5.6%. The average annual temperature and precipitation are about 16 ^◦^C and 1198 mm, respectively. Monthly statistical discharge data from twelve U.S. Geological Survey (USGS) hydrological sites were downloaded from the official website [21]. These monthly statistics generated from sites are based on USGS-approved daily-mean data. The basic information of 12 hydrologic stations and selected descriptive statistics for monthly flow time series are listed in Table 1. Additionally, Figure 2 shows the spatial distribution of meteorological and hydrological stations, terrain, lakes, and rivers in the IRW.

The data used to set up the SWAT model include the following: (1). Digital elevation model (DEM): 1 Arc-Second Global Database from Shuttle Radar Topography Mission (SRTM) were downloaded from the USGS website (https://earthexplorer.usgs.gov/, accessed on 28 January 2018). Its spatial resolution is about 30 m × 30 m. (2). Land use and land cover data: 2011 NLCD dataset (https://www.mrlc.gov/, accessed on 31 January 2018) were applied in this study, and spatial resolution is 100 m × 100 m. (3). Soil data: We downloaded soil data from the SSURGO database (https://websoilsurvey.nrcs.usda.gov/, accessed on 5 February 2018). (4). Climate data: Daily climate data came from the National Climatic Data Center (NCDC) (https://www.ncdc.noaa.gov/, accessed on 7 February 2018). Due to incomplete precipitation and temperature records from January 1990 to December 2013, we downloaded alternative Climate Forecast System Reanalysis (CFSR) data from the SWAT official website (https://globalweather.tamu.edu/, accessed on 31 January 2018), then filled missing NCDC data using climate data from the closest CFSR stations.

### 2.2. Hydrological Model

SWAT was developed by the U.S. Department of Agriculture Agricultural Research Service (USDA-ARS) and has been extensively used worldwide [30,36]. It is a conceptual, semi-distributed, and physically based hydrologic model used to simulate water cycles, crop growth, sediment yields, and agricultural chemical transport in a large river basin with varying soils, slopes, and land use management conditions [30]. A more detailed description of the SWAT model is available from online documentation [37].

ArcSWAT 2012.10_4.19 within ArcGIS 10.4.1 was selected to build the SWAT-based model in the study area. The entire IRW watershed was discretized as 86 subwatersheds with 1023 hydrologic response units (HRUs) using a threshold area of 3000 ha. Each HRU consisted of various land use/soil/slope attributes and was defined with a threshold of land use (10%), soil (10%), and slope (5%). The SCS curve number method [38] and the variable storage routing method [37] were applied to calculate the surface runoff and river flow, respectively. A five-year warm-up period (1990–1994) was set up to initialize the model input and stabilize the SWAT model. The SWAT simulation running period is from 1 January 1995 to 31 December 2013.

We used SWAT-CUP with Sequential Uncertainty Fitting (SUFI2) method to conduct sensitivity analysis, calibration, and validation procedures of the SWAT model [31]. The all-at-a-time approach was applied in the procedure of parameterization with 1000 SWAT-CUP simulations. SWAT-CUP was set up for all twelve stations and run at one time with two iterations. The nine sensitive parameters range were determined and their range and fitted values in calibration listed in Table 2. The results from SWAT-CUP were regarded as a benchmark to compare with results from SWAT-SVR and SWAT-WSVR. The optimal parameters combination and sensitivity of the SWAT model depends on precipitation input, interpolation of weather data, and the number of iterations. It has been investigated in previous publications [11,32,39,40] and will not be discussed further in this article.

### 2.3. Support Vector Machine

SVM is built on the principle of the statistical learning and structural risk minimization theory [41]. When SVM technology is applied in regression analysis, it is called SVR. The SVR function is expressed as below [41]:

(1) f(x)=w⋅Φ(x)+b

where w is a weight vector, Φ is a nonlinear transfer function, and b is offset. An SVR function f(x) can be expressed as the below formulation [17]:

(2) $$\min\frac{1}{2}\|w{\|}^{2}+C\sum_{n=1}^{n}\left(\xi_{i}+\xi_{i}^{*}\right)\;y_{i}-\left(w\cdot \text{Φ}\left(x_{i}\right)+b\right)\leq \varepsilon +\xi_{i}\;\text{subject to}\left(w\cdot \text{Φ}\left(x_{i}\right)+b\right)-y_{i}\leq \varepsilon +\xi_{i}^{*}\;\xi_{i},\xi_{i}^{*}\geq 0,i=1,2,\ldots ,n$$

where $\xi_{i}$ and $\xi_{i}^{*}$ are slack variables that estimate the deviation of training data falling out of the ε-insensitive zone. The C is a penalty factor that determines the tradeoff between the flatness of $\text{Φ}\left(x_{i}\right)$ and the amount up to which the deviation *ε* can be tolerated [42].

In application, SVR includes four commonly used kernel functions such as the linear, polynomial, Gaussian radial basis (RBF), and sigmoid. In this paper, we selected the Gaussian RBF kernel function due to its computational efficiency, and its expression is described below [43]:

(3) $$K\left(x_{i},x_{j}\right)=\exp\left(-\gamma \|x_{i}-x_{j}{\|}^{2}\right)$$

The most critical three parameters in a SVR ε-regression application based on the RBF kernel include: the penalty error parameter C(C>0), the Gaussian RBF kernel parameter γ, and the deviation of the error margin *ε* [14]. We applied the grid search and the *k*-fold cross-validation method to optimize these parameters. The parameters value range in the grid-searching was set up as: *C* (begin = 2^−6^, end = 2^8^, step = 1), γ (begin = 2^4^, end = 2^−8^, step = −1), and ε (begin = 2^−8^, end = 2^−1^, step = 0.5). The *k*-value in cross-validation was set to 5 for tuning the SVR. Before training the SVR, all input data were normalized to the value range [0, 1] by the formula (x−x_min)/(x_max−x_min). Additionally, for each site, we used the first 70% of data to train the model, then applied the remaining 30% subset for validation purposes. The SVR ε-regression model was used to develop both SWAT-SVR and SWAT-WSVR. R version 4.1.0 running on RStudio version 1.4.1717 and the ‘e1071’ package [44] were used for the development, training, and testing of the hybrid model [45].

### 2.4. Wavelet Transforms

The wavelet transform is a mathematical function that has an adjustable time-frequency window and can decompose time series into multiple resolution levels by controlling the scaling and shifting factors of a mother wavelet [46]. A mother wavelet needs to be determined before applying a wavelet analysis. The wavelet transform of time series data generates sets of wavelet coefficients for different scales and provides a time-scale localization of processes [7]. The wavelet transform has two forms: the continuous wavelet transform (CWT) and the discrete wavelet transform (DWT). In this paper, we applied DWT method to build the hybrid SWAT-WSVR model. The DWT discretizes the parameters of scales and positions before implementing the wavelet transform to decrease the redundancy. The DWT of signal f(t) is defined as [47]:

(4) $$W_{f}(j,k)=a_{0}^{-j/2}\int_{-\infty }^{\infty }\psi^{*}\left(a_{0}^{-j}t-kb_{0}\right)f(t)dt$$

where the dilation parameter *a* and temporal translation parameter *b* of the CWT are discretized as $a=a_{0}^{j}$, $b=kb_{0}a_{0}^{j}$, $a_{0}>0$ *and* $a_{0}\neq 1$, $b_{0}\in R$. In most cases, parameter $a_{0}=2$ and $b_{0}=1$. Then, a discrete wavelet can be expressed as [47]:

(5) $$\psi_{j,k}(t)=a_{0}^{-j/2}\psi \left(a_{0}^{-j}t-kb_{0}\right)\;j,k\in Z$$

**Figure 3.**
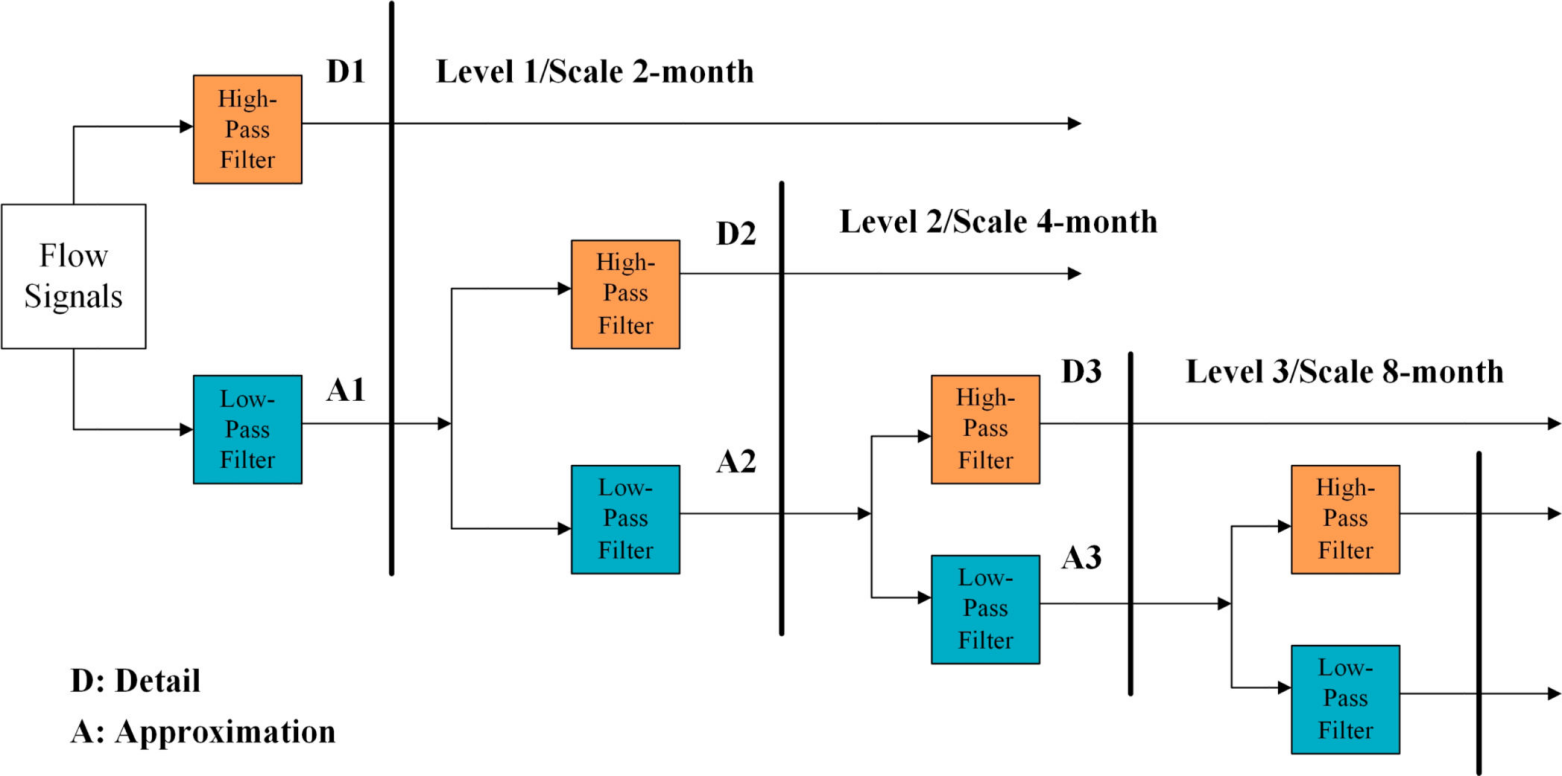
The discrete wavelet transforms (DWT) decomposition procedure of flow time series.

The DWT obtains wavelet details (D) and approximations (A) of the original hydrological time series through high-pass and low-pass filters, respectively. Approximations at various resolution levels can be further decomposed by high-pass and low-pass filters (Figure 3). Commonly used DWT wavelets have ‘Daubechies’, ‘Symlets’, ‘Coiflets’, and ‘Biorthogonal’. More details about wavelet transform can be found in Labat (2005) [48] and Mallat (2009) [49].

We examined Daubechies wavelet family with different wavelengths, such as extremal phase filter with length 1 (‘haar’ or ‘d1’), filter with length 2 (‘d2’), filter with length 4 (‘d4’), filter with length 6 (‘d6’), and Least Asymmetric filter with length 8 (‘la8’), and found that ‘haar’ wavelet is suitable for this study. To date, there is not a standard method to determine the optimum DWT decomposition levels (*D*) for a specific time series. Some applied the equation as D=int(Log(N)) [50]; others used the formula as D=Log(N/(2m−1))/Log(2) [27], where N is the length of monthly time series, and *m* is the number of vanishing moments of a Daubechies wavelet.

In this study, the maximum and minimum length of the monthly flow time series of 12 sites is 228 and 42 (Table 1), respectively. Regardless of applying either the formula mentioned above to calculate *D*, the maximum decomposition levels of monthly flow for all sites were between 1.62 and 7.83. Therefore, wavelet decomposition levels of 1 to 7 were tested to obtain the optimal resolution levels. The results indicated that the decomposition level of 3 and 2 attained the best model performance.

### 2.5. Model Performance Evaluation

We applied four statistics such as *NSE* (Nash–Sutcliffe efficiency), *PBIAS* (percent bias), *RMSE* (root mean square error), and *RSR* (*RMSE*-observation’s standard deviation ratio) to evaluate the model performance in calibration and validation. Table 3 listed these statistical indicators, their mathematic expressions, and their value range. Preferred statistics combination is the lower *RSR*, *PBIAS*, and *RMSE* but the higher *NSE*, which present the better the model prediction performance. We used the ‘hydroGOF’ package [51] in R to calculate the mentioned statistical indicators.

To further compare the performance of SWAT-CUP, SWAT-SVR, and SWAT-WSVR on the entire time series (i.e., combined calibration and validation together), we plotted hydrography for each site and applied the Taylor diagram [52] to examine the relative importance of different statistics such as *r*, *RMSE*, and *NSD* between the observed and simulated flow for three models and twelve sites. The advantage of the Taylor diagram is that it can highlight the goodness-of-fit of multiple models and compare their difference from observed data at the same graph.

## 3. Results and Discussion

We developed a total of 72 models for the flow prediction at 12 sites by three methods: SWAT-CUP, SWAT-SVR, and SWAT-WSVR. Each method included 12 calibrated models and 12 validated models. The difference between SWAT-SVR and SWAT-WSVR is that model inputs of SWAT-SVR had only the flow outputted from SWAT-CUP (a calibrated SWAT model) and precipitation data. Instead, we replaced the simulated flow with its wavelet components at different resolution levels in SWAT-WSVR. Table 4 lists the statistical performance of the three above-mentioned models in calibration, validation, and the whole time series data combining calibration and validation time series data.

### 3.1. Flow Prediction by SWAT-CUP

Table 4 summarizes the average *RSR*, *NSE*, *PBIAS*, and *RMSE* for twelve sites from SWAT-CUP, and the corresponding values are 1.67, 0.22, 57.57, and 11.50 m^3^ s^−1^ in calibration and 0.84, 0.26, 35.98, and 11.04 m^3^ s^−1^ in validation, respectively. The *RSR*, *NSE*, and *RMSE* had approximately similar performances between calibration and validation, but the value of *PBIAS* in validation was lower than one in calibration, which indicated that the predicted discrepancy from validation was less than one from calibration. SWAT-CUP overestimated monthly flow in both calibration and validation. The low average *NSE* value (≤0.26) indicated that SWAT-CUP has a poor goodness-of-fit between the observed and simulated flow for both calibration and validation. Additionally, the 07195430 site had the best performance among all sites in validation with lowest *PBIAS* (−6.7) and *RSR* (0.58) and the highest *NSE* value (0.66). The model performance of 07195500 and 07196500 were also acceptable in validation. After combined calibration and validation data together, the averages of *RSR*, *NSE*, *PBIAS*, and *RMSE* for 12 sites are 0.85, 0.13, 50.67, and 11.38 m^3^ s^−1^, respectively. Simulations from SWAT-CUP greatly overestimated the observed flow according to *PBIAS* (i.e., a positive mean of 50.67 for 12 sites) and presented a low fitting degree due to a low average value (0.13) of *NSE*. Overall, SWAT-CUP had a poor simulation performance for most of sites during both calibration and validation periods, with only few exceptions.

### 3.2. Flow Prediction by SWAT-SVR

Due to the unsatisfactory overall performance of SWAT-CUP, we developed the SWAT-SVR and SWAT-WSVR model integrating the simulated flow (or its wavelet components) and precipitation to improve the prediction accuracy. Table 5 listed the model structure and optimal parameter sets for SWAT-SVR and SWAT-WSVR. In 24 SVR models, the value of C is inconstant from 2.015625 to 255.015625, the value range of γ is from 1 to 14, and ε keeps a constant value of 0.00390625.

The average *RSR*, *NSE*, *PBIAS*, and *RMSE* of twelve sites from SWAT-SVR are 0.58, 0.64, −9.48, and 7.34 m^3^ s^−1^ in calibration and 0.80, 0.32, −20.68, and 11.30 m^3^ s^−1^ in validation (Table 4), respectively. Compared with SWAT-CUP, the performance of the SWAT-SVR model had the lower *RSR* and the absolute value of *PBIAS* and the higher *NSE* in calibration and validation, particularly for the calibrated simulations, as SVR has a strong learning ability for training data. The average *RMSE* in SWAT-SVR calibration is lower: only 7.34 m^3^ s^−1^ compared with the average one of 11.50 m^3^ s^−1^ in the SWAT-CUP calibration. The results showed that the SWAT-SVR calibration on all sites had lower deviation and higher *NSE* value in comparison with SWAT-CUP, but still underestimated monthly flows on most sites. SWAT-SVR was generally superior to SWAT-CUP on all sites. However, only a few sites (e.g., 07195500, 07195430) had lower *RSR*, *PBIAS*, and higher *NSE* values, which indicated that SWAT-SVR could greatly improve the model performance in calibration but did not possess good generalization capability, which means it failed to keep this prediction ability with high accuracy while it was applied in validation. From the perspective of the whole data series, the average *RSR*, *NSE*, *PBIAS*, and *RMSE* are 0.65, 0.57, −13.03, and 8.84 m^3^ s^−1^, respectively. Clearly, compared with SWAT-CUP, the performance of the SWAT-SVR model were improved but limited, although SWAM-SVR generally had a low *RSR*, absolute value of *PBIAS*, and higher *NSE* value for calibration, validation, and both periods.

### 3.3. SWAT-WSVR Development and Evaluation

#### 3.3.1. Development of SWAT-WSVR

**Figure 4.**
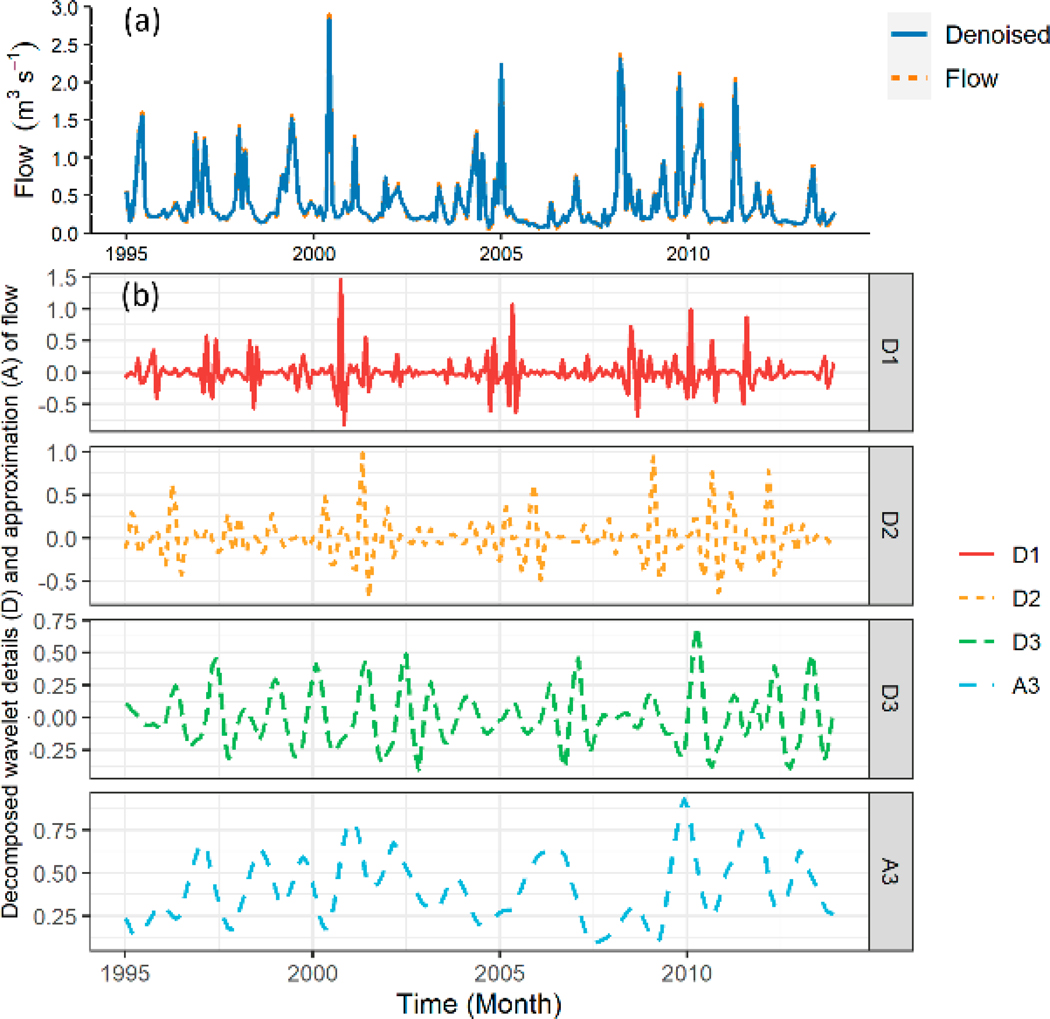
An example of the flow DWT at 07195430 site: (**a**) Observation and SWAT-CUP simulation, (**b**) wavelet decomposition.

We conducted DWT of the simulated flow from SWAT-CUP at twelve hydrological sites using a ‘haar’ wavelet filter to obtain the flow series structure, trend, and temporal characteristics. The wavelet decomposition was implemented at three resolution levels: 2 months, 4 months, and 8 months. Figure 4 showed an example of the flow DWT at the 07195430 site, including temporal features of the flow 2-month mode (D1), 4-month mode (D2), 8-month mode (D3), and approximate mode (A3). From Figure 4a, we can observe a large deviation between the original observed (blue line) and SWAT-CUP simulated (orange dash-line) flow signals. In Figure 4b, D1, D2, and D3 modes indicated high-frequency details, and A3 mode revealed a low-frequency trend and the slowest flow changing of the simulated flow series at the 07195430 site. These wavelet components would be used to build the SWAT-WSVR model afterward.

**Figure 5.**
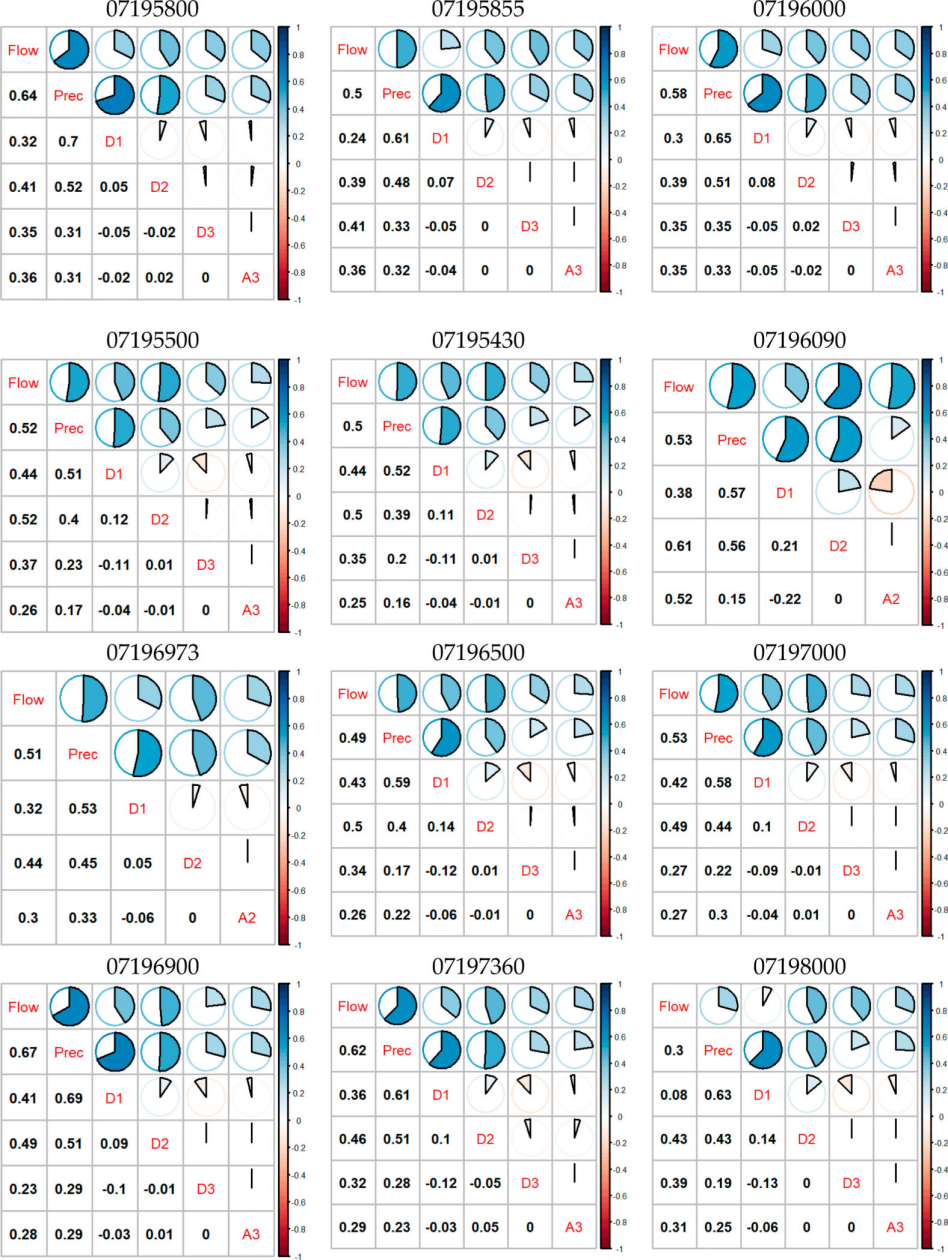
Correlation coefficient matrices of input variables in the SWAT-WSVR model (Note: ‘Prec’ denotes precipitation).

To reduce the computational load and find the most related wavelet coefficients to participate in the construction of SWAT-WSVR, we analyzed Pearson’s correlation coefficient matrices between the observed monthly flow, monthly precipitation, and its sub-time series D1, D2, D3, and A3 from wavelet decompositions (Figure 5). In Figure 5, the upper ‘Pie’ graphs are a corresponding display of the lower ‘numeric’ *r* in the diagonal direction where the flow, precipitation, and wavelet components are shown. Flow time series of 07196090 and 07196973 were decomposed into two resolution levels since their data lengths are shorter: 42 and 96, respectively.

The correlation analysis indicated that the flow and precipitation have a strong positive correlation, and the average value of *r* is 0.53 on twelve sites. Most wavelet components D1, D2, D3, and A3 on the flow also showed positive correlations. The average of *r* of D1, D2, D3, and A3 (or A2) on flow is 0.35, 0.47, 0.3, and 0.32. The wavelet coefficient of D2 had the strongest correlation with the flow. We chose wavelet components with *r* greater than 0.2 to participate in SVR prediction so that we can keep the intrinsic nonlinear features in wavelet components as much as possible while avoiding large computational burden. The specific model structure of SWAT-WSVR for each site is listed in Table 5.

#### 3.3.2. Statistical Evaluation of SWAT-WSVR

Table 4 showed the statistical performance of SWAT-WSVR with the average *RSR*, *NSE*, *PBIAS*, and *RMSE* of 0.02, 1.00, −0.15, and 0.27 m^3^ s^−1^ in calibration; 0.14, 0.98, −1.88, and 2.91 m^3^ s^−1^ in validation; and 0.08, 0.99, −0.74, and 1.61 m^3^ s^−1^ in the whole data series. Compared with SWAT-SVR and SWAT-CUP, SWAT-WSVR had the lowest *RSR*, the absolute value of *PBIAS*, and *RMSE* but the highest *NSE* value in validation. This result clearly indicated that SWAT-WSVR could effectively decrease the discrepancy of the simulation and obtain the best prediction accuracy for validation in comparison with SWAT-SVR and SWAT-CUP. Based on the value of *PBIAS*, SWAT-WSVR slightly underestimated the monthly flow in calibration. SWAT-WSVR also presented the best performance on the whole data series, along with the lowest *RSR*, *PBIAS*, and *RMSE* but the highest *NSE* in comparison with SWAT-CUP and SWAT-SVR. By comparison, the SWAT-WSVR model outperformed the SWAT-CUP and SWAT-SVR model in calibration, validation, and both periods.

### 3.4. Taylor Diagram and Hydrographic Comparison between Different Models

**Figure 6.**
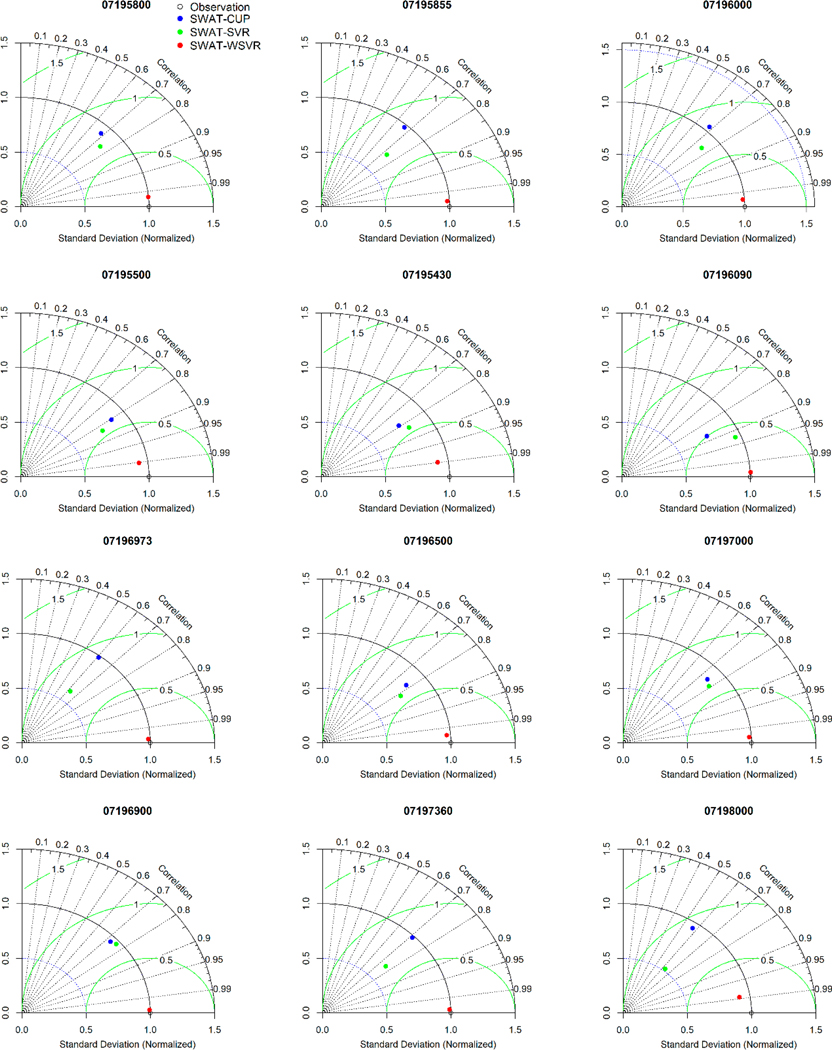
Taylor diagram of three models in comparison with the observation for each site.

To further compare the overall performance of three models, we combined the flow observations and the calibrated and validated simulations from SWAT-CUP, SWAT-SVR, and SWAT-WSVR; recalculated statistical indicators including *r*, *RMSE*, and *NSD*; and re-estimated the model performance on the whole time series (i.e., calibration and validation periods are considered together). The Taylor diagrams (Figure 6) depicted the overall performance of different models for each site by identifying the pattern correlations, variability, and *RMSE* between observations and simulations. In Figure 6, both the x-axis and y-axis denote *NSD*; black dashed lines represent the *r* between observations and simulations; the normalized *RMSE* of the simulation is proportional to the distance from the *x*-axis identified as “observation” (green contours); the *NSD* of the simulation is proportional to the radial distance from the origin point (black contours). The modeling results in Figure 6 demonstrated that the developed SWAT-WSVR had the best performance in comparison with the other two models since it had the lower *RMSE* and *NSD*, but the higher *r* with observed flows in most cases. For example, SWAT-WSVR (redpoint in Taylor diagram) at all sites is closer to the reference point (observation) located on the *x*-axis than the other two models, which illustrated the SWAT-WSVR fitted best with the observed flow for most sites. The rank of the overall performance of three models from high to low follows SWAT-WSVR > SWAT-SVR > SWAT-CUP. The proposed SWAT-WSVR model that uses wavelet components as inputs of SVR presented a more satisfactory flow prediction than the SWAT-SVR with a single pattern input (i.e., the simulated flow from SWAT-CUP). Possible reasons include a periodical feature of sub-series represented by wavelet components is more obvious than those directly obtained from SWAT-CUP [53], and SVR captured the intrinsic nonlinear features between SWAT-CUP simulations and observed flows and built a mapping relationship at a higher dimension space successfully. This kind of fitting relationship is typically determined by using the trial-and-error method and a large number of iterations of many parameters sets in physically based hydrological models.

**Figure 7.**
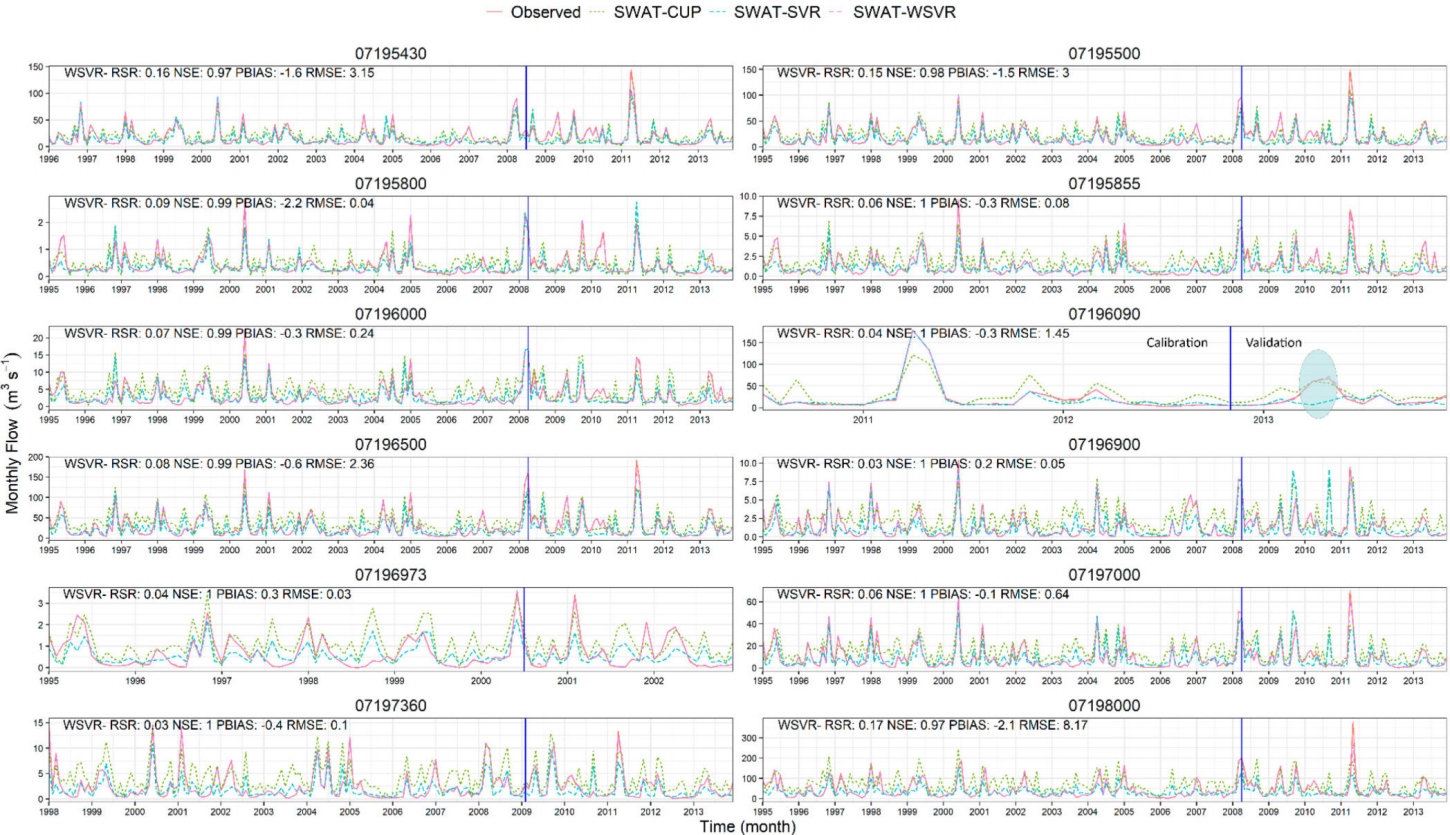
Comparison of SWAT-CUP, SWAT-SVR, SWAT-WSVR, and observed flow time series on each site. (Note: a vertical blue line separated the 70% training and 30% testing data. Only statistics from SWAT-WSVR were labeled for clarity).

To investigate the entire and continuous performance of monthly flow prediction in the IRW, we plotted flow hydrography on the whole data series for each site (Figure 7). Here, we only labeled statistics of SWAT-WSVR for clarity, and other details related to estimate indicators can be found in Table 4. This figure reflected where the developed SWAT-WSVR model performed better than SWAT-CUP and SWAT-SVR methods. An oval region at the 07196090 site showed clear evidence that SWAT-WSVR agreed well with the observed flow, but SWAT-SVR missed the peak flow during this time window. Although SWAT-WSVR had desirable modeling performance, it missed few peak flows. For example, the SWAT-WSVR simulated flow (105.24 and 107.68 m^3^ s^−1^) was 27.2% and 27.9% lower than observed flow (144.61 and 149.42 m^3^ s^−1^) in April 2011 at 07195430 and 07195500 site, respectively. Overall, the other two models more or less capture the rising and recession of the observed monthly flow over time at all sites, but the SWAT-WSVR is more efficient at fitting with the observation and corrected errors compared to SWAT-CUP. This result is in line with others’ conclusions that the application of wavelet transform in data-driven models can improve the accuracy of flow prediction [53,54]. The developed SWAT-WSVR model fit the observations well at all sites of the IRW based on the statistical results, Taylor diagram, and hydrography analysis.

In this study, we applied wavelet transforms on the simulated flow time series from SWAT-CUP rather than on the observed flow data to show that the developed SWAT-WSVR model can be applied in practice or future scenario prediction where observed data are impossible to access or has limited availability. An SVR coupled to a wavelet transform model approach based solely on observed monthly flow data could produce greater accuracy. This is because wavelet decompositions with less noise can represent the periodical and trend characteristics of flow series structure better than the original data series [25,53], and SVR has a strong learning capability to capture the corresponding relationship between wavelet decompositions and its original data series. However, the limitations of a purely mathematical or machine learning approach prior to constraint by a physically-based model could fail to capture the relationship between rainfall and runoff and exhibit less predictive or unrealistic behavior [28].

Moreover, the current proposed SWAT-WSVR model can be regarded as a compromise method when we cannot attain a desirable prediction accuracy even after conducting extensive SWAT-CUP iterative computation, although we still encourage researchers to directly obtain a satisfactory prediction from SWAT-CUP if possible. In this work, the construction of SWAT-WSVR heavily depends on the procedure of SWAT-CUP parameter calibration. Alternatively, we can also build SWAT-WSVR based on wavelet decomposition of the initial output from SWAT without the procedure of calibration based on the methodology proposed if SWAT-CUP is not accessible.

## 4. Summary and Conclusions

This study developed the SWAT-WSVR monthly flow prediction model which was built on the basis of the SWAT and SVR with discrete wavelet transforms and investigated the performance and effectiveness of this model. Precipitation and wavelet components of flow outputted from SWAT-CUP were served as input variables into SWAT-WSVR. The methodology loosely integrated the physically based model and the data-driven model. The proposed SWAT-WSVR model had the best statistical performance with the lower *RSR*, the absolute value of *PBIAS*, *RMSE*, and higher *NSE* in comparison with regular SWAT-SVR and SWAT-CUP, which indicated that SWAT-WSVR possessed the lower discrepancy and higher goodness-of-fit between the simulated and observed flow. The rank of the overall performance of the three models on the entire study period was SWAT-WSVR > SWAT-SVR > SWAT-CUP. The SWAT-WSVR model can predict monthly flow more accurately than the other two models for all sites in the IRW.

The strength of the SWAT-WSVR is its ability to capture the intrinsic nonlinear and non-stationary features between rainfall and runoff while considering physical processes by integrating SWAT. It could be regarded as a compromise method when one cannot directly obtain a desirable accuracy from SWAT-CUP simulation or when one applies SWAT into a region with limited data available. In future work, more predictors (e.g., temperature, evaporation, relative humidity) will be considered as the model input variables to raise the forecasting accuracy of SWAT-WSVR further and increase its generalization ability.

## Figures and Tables

**Table 1.** Watershed properties and selected descriptive statistics of USGS hydrological stations.

No.	USGS Station	Upstream Area (km^2^)	Data Period (month.year)	Number of Data	Average Monthly Streamflow (m^3^ s^−1^)	Flow Descriptive Statistics (m^3^ s^−1^)			
						Max	Min	Median	Standard Deviation

1	07195800	36.8	1.1995–12.2013	228	0.41	2.90	0.05	0.25	0.44
2	07195855	155.0	1.1995–12.2013	228	1.27	9.53	0.11	0.74	1.43
3	07196000	300.7	1.1995–12.2013	228	3.01	22.26	0.42	1.78	3.25
4	07195500	1633.0	1.1995–12.2013	228	18.71	149.42	2.73	10.75	20.02
5	07195430	1490.5	1.1996–12.2013	216	17.68	144.61	1.89	10.58	19.29
6	07196090	2138.5	7.2010–12.2013	42	23.19	178.54	2.95	11.77	33.59
7	07196973	64.8	1.1995–12.2002	96	0.66	3.57	0.00	0.38	0.75
8	07196500	2462.5	1.1995–12.2013	228	27.76	190.80	2.99	17.09	30.17
9	07197000	808.7	1.1995–12.2013	228	9.27	69.73	0.33	4.93	11.44
10	07196900	105.2	1.1995–12.2013	228	1.31	10.35	0.00	0.59	1.76
11	07197360	233.8	1.1998–12.2013	192	2.41	15.18	0.10	1.46	2.88
12	07198000	4186.2	1.1995–12.2013	228	44.03	378.65	0.98	25.59	46.81

**Table 2.** The sensitive parameters range and their fitted values in calibration.

No.	Parameter Name ^†^	Parameter Description	Range	Fitted Value

1	R_CN2.mgt	SCS runoff curve number II	−0.25–0.25	−0.179
2	V_GWQMN.gw	Threshold depth of water in the shallow aquifer required for return flow to occur (mm H_2_O)	0–2000	1764
3	V_GW_REVAP.gw	Groundwater “revap” coefficient	0.02–0.2	0.135
4	V_REVAPMN.gw	Threshold depth of water in the shallow aquifer for ‘revap’ to occur (mm)	0–500	121
5	V_EPCO.hru	Plant uptake compensation factor	0–1	0.154
6	V_ESCO.hru	Soil evaporation compensation factor	0–1	0.354
7	R_SOL_AWC (1).sol	Available water capacity of the 1st soil layer (mm H_2_O mm soil^−1^)	0.08–0.2	0.177
8	A_OV_N.hru	Manning’s “n” value for overland flow	0.01–30	26.941
9	R_HRU_SLP.hru	Average slope steepness (m m^−1^)	0–1	0.034

Note: “A_”, “V_”, and “R_” mean an absolute increase, a replacement, and a relative change to the initial parameter values, respectively.

**Table 3.** Evaluation indicators of the model performance and their mathematic expressions.

Indicator Name	Calculation Equation ^†^	Description

**Pearson’s Correlation Coefficient (*r*)**	$r=\frac{n\left(\sum yy_{i}\right)-\left(\sum y\right)\left(\sum y_{i}\right)}{\sqrt{\left[n\sum y^{2}-{\left(\sum y\right)}^{2}\right]\left[n\sum y^{\prime }-{\left(\sum y^{\prime }\right)}^{2}\right]}}$	Range [−1, 1]
**Nash-Sutcliffe efficiency (*NSE*)**	$NSE=1-\frac{{\sum_{i=1}^{n}\left(y_{i}-y_{i}^{\prime }\right)}^{2}}{\sum_{i=1}^{n}{\left(y_{i}-\bar{y}\right)}^{2}}$	Range (−∞, 1], and 1 is the optimal value
**Percent Bias (*PBIAS*)**	$PBIAS=100\times \frac{\sum_{i=1}^{n}\left({y_{i}}^{\prime }-y_{i}\right)}{\sum_{i=1}^{n}y_{i}}$	Range (−∞, +∞), and 0 is the optimal value
**RMSE-observations standard deviation ratio (*RSR*)**	$RSR=\frac{\sqrt{\sum_{i=1}^{n}(y_{i}-{y_{i}}^{\prime }{)}^{2}}}{\sqrt{\sum_{i=1}^{n}(y_{i}-\bar{y}{)}^{2}}}$	Range [0, +∞), and 0 is the optimal value
**Root Mean Square Error (*RMSE*)**	$RMSE=\sqrt{\frac{\sum_{i=1}^{n}{\left(y_{i}-y_{i}^{\prime }\right)}^{2}}{n}}$	Range [0, +∞), and 0 is the optimal value

Note: $y_{i}$is the observed data series, $y_{i}^{\prime }$is the simulated results series, the overbar represents the mean value of data series, and *n* is the sample number.

**Table 4.** Performance of flow calibration, validation, and combined data series on each site by SWAT-CUP, SWAT-SVR, and SWAT-WSVR.

Station		SWAT-CUP				SWAT-SVR				SWAT-WSVR			

		*RSR*	*NSE*	*PBIAS*	*RMSE* (m^3^ s^−1^)	*RSR*	*NSE*	*PBIAS*	*RMSE* (m^3^ s^−1^)	*RSR*	*NSE*	*PBIAS*	*RMSE* (m^3^ s^−1^)

Calibration	07195800	0.76	0.41	24.00	0.33	0.58	0.68	−10.80	0.25	0.08	0.99	−1.90	0.04
	07195855	0.95	0.09	66.50	1.32	0.63	0.60	−10.00	0.88	0.01	1.00	0.10	0.01
	07196000	0.96	0.07	67.90	3.18	0.57	0.68	0.50	1.88	0.01	1.00	0.10	0.02
	07195500	0.74	0.46	44.60	13.68	0.55	0.70	−5.50	10.23	0.01	1.00	0.10	0.23
	07195430	0.67	0.54	29.30	11.40	0.55	0.70	−5.30	9.30	0.01	1.00	0.20	0.22
	07196090	0.52	0.72	31.40	19.81	0.14	0.98	−3.30	5.18	0.02	1.00	−0.60	0.70
	07196973	10.70	−0.15	69.90	0.78	0.73	0.46	−6.50	0.53	0.02	1.00	0.20	0.01
	07196500	0.78	0.39	52.30	22.62	0.58	0.67	−10.20	16.71	0.02	1.00	0.10	0.48
	07197000	0.87	0.24	69.50	10.04	0.57	0.67	−11.80	6.64	0.01	1.00	0.10	0.09
	07196900	0.93	0.14	86.00	1.69	0.57	0.68	−13.60	1.04	0.01	1.00	0.00	0.02
	07197360	0.97	0.06	74.90	2.89	0.70	0.51	−21.30	2.07	0.00	1.00	0.00	0.01
	07198000	1.17	−0.39	74.50	50.21	0.78	0.39	−15.90	33.42	0.03	1.00	−0.20	1.41
	**Mean**	**1.67**	**0.22**	**57.57**	**11.50**	**0.58**	**0.64**	**-9.48**	**7.34**	**0.02**	**1.00**	**-0.15**	**0.27**

Validation	07195800	0.85	0.26	8.70	0.35	0.89	0.20	−20.40	0.36	0.11	0.99	−2.90	0.04
	07195855	0.89	0.19	22.80	1.35	0.81	0.33	−27.00	1.23	0.09	0.99	−1.10	0.14
	07196000	0.98	0.03	29.60	3.03	0.86	0.24	−16.00	2.68	0.14	0.98	−1.00	0.43
	07195500	0.59	0.65	15.40	13.59	0.58	0.65	−20.50	13.48	0.24	0.94	−4.60	5.49
	07195430	0.58	0.66	−6.70	13.88	0.58	0.68	−23.80	13.74	0.24	0.94	−4.80	5.77
	07196090	0.71	0.45	38.30	14.32	1.15	−0.43	−41.70	23.16	0.12	0.98	0.70	2.47
	07196973	1.03	−0.10	62.30	0.83	0.87	0.22	−15.70	0.69	0.06	1.00	0.70	0.05
	07196500	0.66	0.56	19.70	21.55	0.63	0.60	−25.50	20.62	0.13	0.98	−2.20	4.28
	07197000	0.88	0.22	64.80	9.84	0.72	0.47	−6.30	8.07	0.10	0.99	−0.40	1.17
	07196900	1.07	−0.17	87.90	1.72	0.96	0.06	3.60	1.54	0.05	0.99	0.60	0.08
	07197360	0.88	0.21	55.80	2.34	0.66	0.58	−25.10	1.76	0.07	0.99	−1.40	0.18
	07198000	0.90	0.18	33.20	49.62	0.84	0.29	−29.80	48.22	0.27	0.93	−6.10	14.81
	**Mean**	**0.84**	**0.26**	**35.98**	**11.04**	**0.80**	**0.32**	**-20.68**	**11.30**	**0.14**	**0.98**	**-1.88**	**2.91**

The whole series data ^†^	07195800	0.79	0.37	19.50	0.33	0.68	0.53	−13.60	0.29	0.09	0.99	−2.20	0.04
	07195855	0.93	0.13	52.30	1.33	0.70	0.51	−15.50	1.00	0.06	1.00	−0.30	0.08
	07196000	0.97	0.06	55.90	3.13	0.66	0.56	−4.60	2.15	0.07	0.99	−0.30	0.24
	07195500	0.68	0.53	35.00	13.65	0.56	0.68	−10.40	11.30	0.15	0.98	−1.50	3.00
	07195430	0.63	0.60	16.80	12.19	0.56	0.68	−11.70	10.81	0.16	0.97	−1.60	3.15
	07196090	0.55	0.69	33.50	18.41	0.39	0.84	−14.70	13.13	0.04	1.00	−0.30	1.45
	07196973	1.06	−0.14	67.80	0.79	0.78	0.38	−9.10	0.58	0.04	1.00	0.30	0.03
	07196500	0.74	0.45	41.80	22.31	0.60	0.64	−15.10	17.97	0.08	0.99	−0.60	2.36
	07197000	0.87	0.24	68.20	9.98	0.62	0.61	−10.30	7.90	0.06	1.00	−0.10	0.64
	07196900	0.97	0.06	86.50	1.70	0.69	0.53	−8.70	1.21	0.03	1.00	0.20	0.05
	07197360	0.95	0.10	69.50	2.73	0.69	0.52	−22.40	1.98	0.03	1.00	−0.40	0.10
	07198000	1.07	−1.50	61.20	50.03	0.81	0.35	−20.30	37.70	0.17	0.97	−2.10	8.17
	**Mean**	**0.85**	**0.13**	**50.67**	**11.38**	**0.65**	**0.57**	**−13.03**	**8.84**	**0.08**	**0.99**	**−0.74**	**1.61**

† Note: The data series combined calibration and validation time series.

**Table 5.** Model inputs and optimum parameters of SWAT-SVR and SWAT-WSVR.

Station	SWAT-SVR			SWAT-WSVR			

	Model Input ^†^	*C*	*γ*	Model Input	Decomposition Levels	*C*	*γ*

07195800	Flow + Prec	36.015625	3	Prec + D1 + D2 + D3 + A3	3	5.015625	1
07195855	Flow + Prec	22.015625	1	Prec + D1 + D2 + D3 + A3	3	2.015625	3
07196000	Flow + Prec	255.015625	1	Prec + D1 + D2 + D3 + A3	3	255.015625	1
07195500	Flow + Prec	103.015625	1	Prec + D1 + D2 + D3 + A3	3	96.015625	1
07195430	Flow + Prec	255.015625	1	Prec + D1 + D2 + D3 + A3	3	87.015625	1
07196090	Flow + Prec	255.015625	5	Prec + D1 + D2 + A2	2	255.015625	1
07196973	Flow + Prec	2.015625	1	Prec + D1 + D2 + A2	2	5.015625	1
07196500	Flow + Prec	130.015625	1	Prec + D1 + D2 + D3 + A3	3	125.015625	1
07197000	Flow + Prec	57.015625	1	Prec + D1 + D2 + D3 + A3	3	242.015625	1
07196900	Flow + Prec	4.015625	14	Prec + D1 + D2 + D3 + A3	3	15.015625	1
07197360	Flow + Prec	13.015625	1	Prec + D1 + D2 + D3 + A3	3	35.015625	1

† Note: Flow comes from SWAT-CUP simulated discharge output. Ds and As are wavelet components from the simulated flow of SWAT-CUP.

## Data Availability

https://doi.org/10.23719/1527817.

## References

[1] Alizadeh MJ; Kavianpour MR; Kisi O; Nourani V. A new approach for simulating and forecasting the rainfall-runoff process within the next two months. J. Hydrol 2017, 548, 588–597.

[2] Huo Z; Feng S; Kang S; Huang G; Wang F; Guo P. Integrated neural networks for monthly river flow estimation in arid inland basin of Northwest China. J. Hydrol 2012, 420–421, 159–170.

[3] U.S. EPA. A Review of Watershed and Water Quality Tools for Nutrient Fate and Transport; EPA/600/R-19/232; Center for Environmental Solutions & Emergency Response|Groundwater Characterization & Remediation Division, Office of Research and Development (EPA): Ada, OK, USA, 2019.

[4] Kalteh AM Monthly River Flow Forecasting Using Artificial Neural Network and Support Vector Regression Models Coupled with Wavelet Transform. Comput. Geosci 2013, 54, 1–8.

[5] Yuan L; Zhou Q. Complexity of soil erosion and sediment yield system in a watershed. J. Chongqing Inst. Technol. (Nat. Sci.) 2008, 22, 112–116.

[6] Zhang Z; Chen X; Xu C; Yuan L; Yong B; Yan S. Evaluating the non-stationary relationship between precipitation and streamflow in nine major basins of China during the past 50 years. J. Hydrol 2011, 409, 81–93.

[7] Nourani V; Hosseini Baghanam A; Adamowski J; Kisi O. Applications of hybrid wavelet–Artificial Intelligence models in hydrology: A review. J. Hydrol 2014, 514, 358–377.

[8] Yuan L; Yang G; Li H; Zhang Z. Spatio-Temporal Variation Analysis of Precipitation during 1960–2008 in the Poyang Lake Basin, China. Open J. Mod. Hydrol 2016, 6, 115–127.

[9] Kratzert F; Klotz D; Herrnegger M; Sampson AK; Hochreiter S; Nearing GS Toward improved predictions in ungauged basins: Exploiting the power of machine learning. Water Resour. Res 2019, 55, 11344–11354.

[10] Hsu K.l.; Gupta HV; Sorooshian S. Artificial neural network modeling of the rainfall-runoff process. Water Resour. Res 1995, 31, 2517–2530.

[11] Yuan L; Forshay K. Enhanced streamflow prediction with SWAT using support vector regression for spatial calibration: A case study in the Illinois River watershed, U.S. PLoS ONE 2021, 16, e0248489. 10.1371/journal.pone.0248489 PMC8041176 33844687

[12] Smola AJ; Schölkopf B. A tutorial on support vector regression. Stat. Comput 2004, 14, 199–222.

[13] Noraini I; Norhaiza A. Comparative performance of support vector regressions for accurate streamflow predictions. Malays. J. Fundam. Appl. Sci 2017, 325–330.

[14] Misra D; Oommen T; Agarwal A; Mishra SK; Thompson AM Application and Analysis of Support Vector Machine Based Simulation for Runoff and Sediment Yield. Biosyst. Eng 2009, 103, 527–535.

[15] Zhang X; Srinivasan R; Van Liew M. Approximating SWAT model using artificial neural network and support vector machine. J. Am. Water Resour. Assoc 2009, 45, 460–474.

[16] Yuan L; Li W; Zhang Q; Zou L. Debris flow hazard assessment based on support vector machine. In Proceedings of the IEEE International Symposium on Geoscience and Remote Sensing, Denver, CO, USA, 31 July–4 August 2006; pp. 4221–4224.

[17] Raghavendra NS; Deka PC Support vector machine applications in the field of hydrology: A review. Appl. Soft Comput 2014, 19, 372–386.

[18] Shabri A; Suhartono. Streamflow forecasting using least-squares support vector machines. Hydrol. Sci. J 2012, 57, 1275–1293.

[19] Chiogna G; Marcolini G; Liu W; Perez Ciria T; Tuo Y. Coupling hydrological modeling and support vector regression to model hydropeaking in alpine catchments. Sci. Total Environ 2018, 633, 220–229. 29573688 10.1016/j.scitotenv.2018.03.162

[20] Nourani V; Alizadeh F; Roushangar K. Evaluation of a two-stage SVM and spatial statistics methods for modeling monthly river suspended sediment load. Water Resour. Manag 2015, 30, 393–407.

[21] USGS Water Data for the Nation. Available online: https://nwis.waterdata.usgs.gov (accessed on 18 January 2018).

[22] Hrachowitz M; Savenije HHG; Blöschl G; McDonnell JJ; Sivapalan M; Pomeroy JW; Arheimer B; Blume T; Clark MP; Ehret U; A decade of Predictions in Ungauged Basins (PUB)—A review. Hydrol. Sci. J 2013, 58, 1198–1255.

[23] Devia GK; Ganasri BP; Dwarakish GS A Review on Hydrological Models. Aquat. Procedia 2015, 4, 1001–1007.

[24] Liu Z; Zhou P; Chen G; Guo L. Evaluating a coupled discrete wavelet transform and support vector regression for daily and monthly streamflow forecasting. J. Hydrol 2014, 519, 2822–2831.

[25] Zhu S; Zhou J; Ye L; Meng C. Streamflow estimation by support vector machine coupled with different methods of time series decomposition in the upper reaches of Yangtze River, China. Environ. Earth Sci 2016, 75, 531.

[26] Sun Y; Niu J; Sivakumar B. A comparative study of models for short-term streamflow forecasting with emphasis on wavelet-based approach. Stoch. Environ. Res. Risk Assess 2019, 33, 1875–1891.

[27] Nalley D; Adamowski J; Khalil B; Biswas A. A comparison of conventional and wavelet transform based methods for streamflow record extension. J. Hydrol 2020, 582, 124503.

[28] Quilty J; Adamowski J. Addressing the incorrect usage of wavelet-based hydrological and water resources forecasting models for real-world applications with best practices and a new forecasting framework. J. Hydrol 2018, 563, 336–353.

[29] Yuan L; Sinshaw T; Forshay KJ Review of watershed-scale water quality and nonpoint source pollution models. Geosciences 2020, 10, 25. 10.3390/geosciences10010025 PMC7513854 32983579

[30] Arnold JG; Moriasi DN; Gassman PW; Abbaspour KC; White MJ; Srinivasan R; Santhi C; Harmel R; Van Griensven A; Van Liew MW SWAT: Model use, calibration, and validation. Trans. ASABE 2012, 55, 1491–1508.

[31] Abbaspour KC SWAT-CUP: SWAT Calibration and Uncertainty Programs—A User Manual; Eawag: Swiss Federal Institute of Aquatic Science and Technology: Zurich, Switzerland, 2015.

[32] Yuan L; Forshay KJ Using SWAT to evaluate streamflow and lake sediment loading in the Xinjiang River basin with limited data. Water 2019, 12, 39. 32983578 10.3390/w12010039 PMC7513863

[33] Jajarmizadeh M; Kakaei Lafdani E; Harun S; Ahmadi A. Application of SVM and SWAT models for monthly streamflow prediction, a case study in South of Iran. KSCE J. Civ. Eng 2014, 19, 345–357.

[34] Jimeno-Sáez P; Senent-Aparicio J; Pérez-Sánchez J; Pulido-Velazquez D. A comparison of SWAT and ANN models for daily runoff simulation in different climatic zones of peninsular Spain. Water 2018, 10, 192.

[35] Noori N; Kalin L. Coupling SWAT and ANN models for enhanced daily streamflow prediction. J. Hydrol 2016, 533, 141–151.

[36] Gassman PW; Balmer C; Siemers M; Srinivasan R. The SWAT literature database: Overview of database structure and key SWAT literature trends. In Proceedings of the SWAT 2014 Conference, Pernambuco, Brazil, 28 July–1 August 2014. Available online: http://swat.tamu.edu/conferences/2014/ (accessed on 27 June 2018).

[37] Neitsch SL; Arnold JG; Kiniry JR; Williams JR Soil and Water Assessment Tool Theoretical Documentation Version 2009; TR-406; Texas Water Resources Institute: College Station, TX, USA, 2009.

[38] Soil Conservation Service. National Engineering Handbook; United States Department of Agriculture: Washington, DC, USA, 1972; Section 4 Hydrology.

[39] Storm DE; Busteed PR; Mittelstet AR; White MJ Hydrologic Modeling of the Oklahoma/Arkansas Illinois River Basin Using SWAT 2005; Oklahoma Department of Environmental Quality: Stillwater, OK, USA, 2010.

[40] Mittelstet AR; Storm DE; White MJ Using SWAT to enhance watershed-based plans to meet numeric water quality standards. Sustain. Water Qual. Ecol 2016, 7, 5–21.

[41] Vapnik V. *The Nature of Statistical Learning Theory*; Springer: New York, NY, USA, 1995.

[42] Danandeh Mehr A; Nourani V; Karimi Khosrowshahi V; Ghorbani MA A hybrid support vector regression–firefly model for monthly rainfall forecasting. Int. J. Environ. Sci. Technol 2018, 16, 335–346.

[43] Hsu C-W; Chang C-C; Lin C-J *A Practical Guide to Support Vector Classification*; Department of Computer Science, National Taiwan University: Taipei, Taiwan, 2003.

[44] Meyer D; Dimitriadou E; Hornik K; Weingessel A; Leisch F. e1071: Misc Functions of the Department of Statistics, Probability Theory Group (Formerly: E1071); Rstudio: Boston, MA, USA, 2019.

[45] R Core Team. R: A Language and Environment for Statistical Computing; Rstudio: Boston, MA, USA, 2022.

[46] Liu Z; Menzel L. Identifying long-term variations in vegetation and climatic variables and their scale-dependent relationships: A case study in Southwest Germany. Glob. Planet. Chang 2016, 147, 54–66.

[47] Makwana JJ; Tiwari MK Intermittent Streamflow Forecasting and Extreme Event Modelling using Wavelet based Artificial Neural Networks. Water Resour. Manag 2014, 28, 4857–4873.

[48] Labat D. Recent advances in wavelet analyses: Part 1. A review of concepts. J. Hydrol 2005, 314, 275–288.

[49] Mallat S. A Wavelet Tour of Signal Processing; Elsevier: Amsterdam, The Netherlands, 2009.

[50] Nourani V; Komasi M; Mano A. A multivariate ANN-Wavelet approach for rainfall–runoff modeling. Water Resour. Manag 2009, 23, 2877–2894.

[51] Zambrano-Bigiarini M. hydroGOF: Goodness-of-Fit Functions for Comparison of Simulated and Observed Hydrological Time Series; Rstudio: Boston, MA, USA, 2017.

[52] Taylor KE Summarizing multiple aspects of model performance in a single diagram. J. Geophys. Res. Atmos 2001, 106, 7183–7192.

[53] Kisi O; Cimen M. A wavelet-support vector machine conjunction model for monthly streamflow forecasting. J. Hydrol 2011, 399, 132–140.

[54] De Macedo Machado Freire PK; Santos CAG; da Silva GBL. Analysis of the use of discrete wavelet transforms coupled with ANN for short-term streamflow forecasting. Appl. Soft Comput 2019, 80, 494–505.

